# Supplementary material for: Single‐cell dynamics of chromatin activity during cell lineage differentiation in Caenorhabditis elegans embryos
Source: Mol Syst Biol. 2021 Apr 26;17(4):e10075. doi: 10.15252/msb.202010075 (PMC8073016; doi:10.15252/msb.202010075)
Supplement: Supplementary file 2 — Appendix [file MSB-17-e10075-s001.pdf]

Appendix

Table of Contents

Appendix Fig S1. Position effects on GFP expression at 113 genomic positions in 364 traced terminal cells. 3

Appendix Fig S2. Expression of P*eef-1A.1*::GFP exhibits strong variability between experimental replicates at certain integration sites. 5

Appendix Fig S3. Chromatin activity landscape is coupled to tissue fate. 6

Appendix Fig S4. Chromatin activity landscape is associated with tissue fate after controlling for the influence of cell lineage. 8

Appendix Fig S5. Predetermination of chromatin activity landscape during L-R symmetry establishment. 9

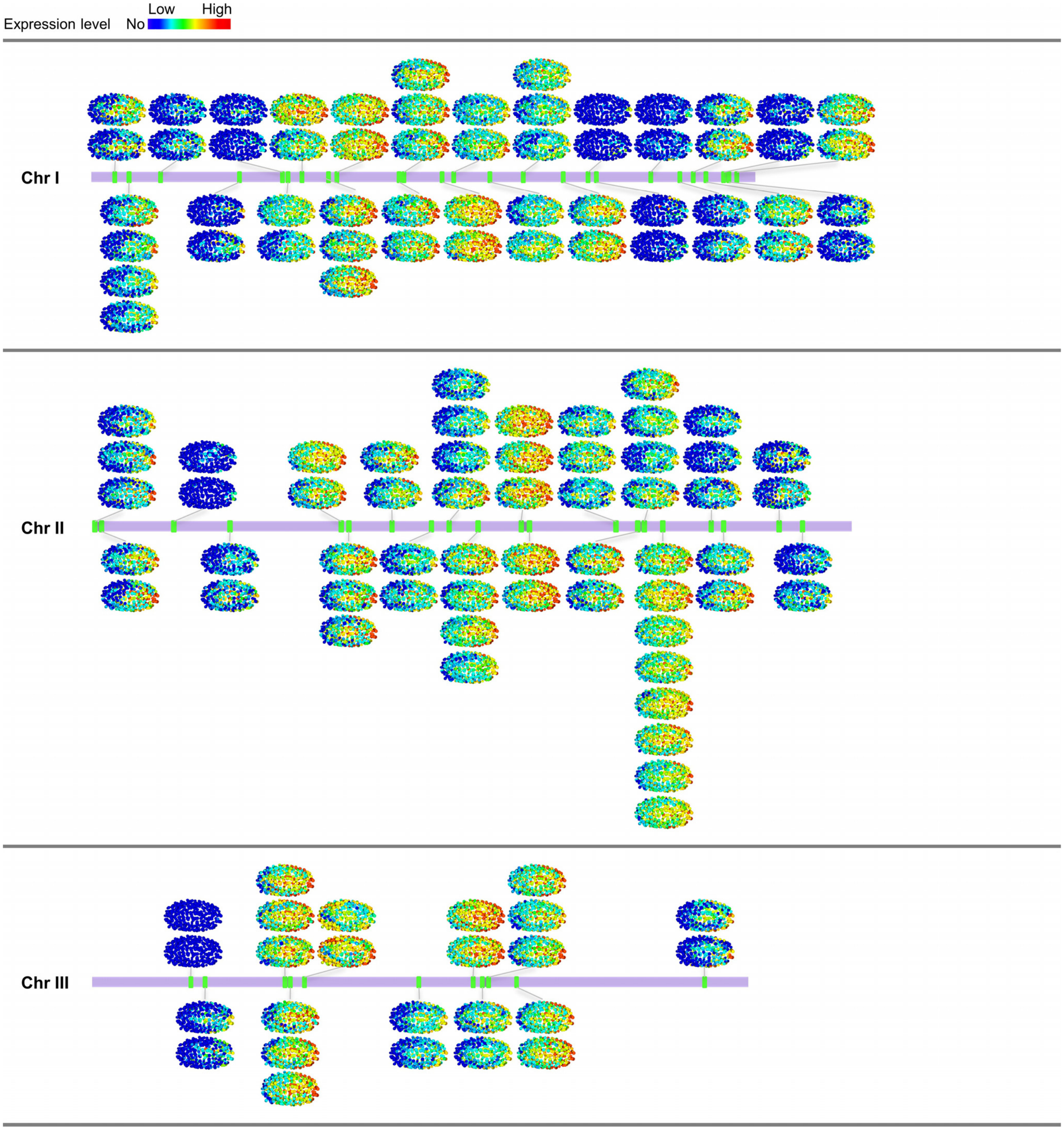
Expression level
No
Low
High
Chr I
Chr II
Chr III

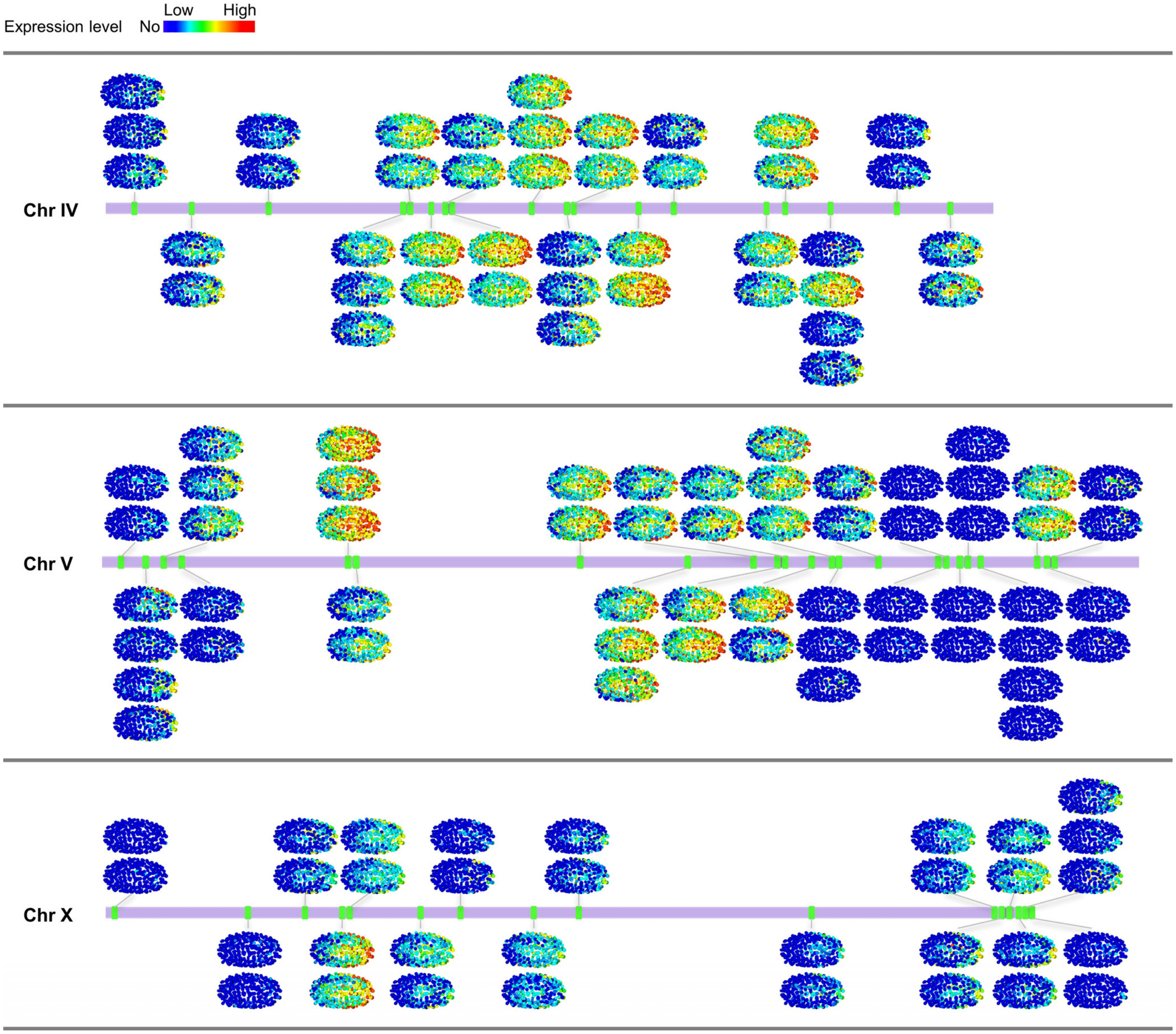

Appendix Fig S1. Position effects on GFP expression at 113 genomic positions in 364 traced terminal cells.

3D visualization of cellular expression of GFP integrated into 113 genomic regions in 268 analyzed embryos. Each ellipsoid is a 3D rendering of a standardized embryo, with dots indicating cells and the color gradient indicating GFP expression level. Green vertical lines indicate the locations of the integration sites in the genome. Multiple experimental replicates are stacked.

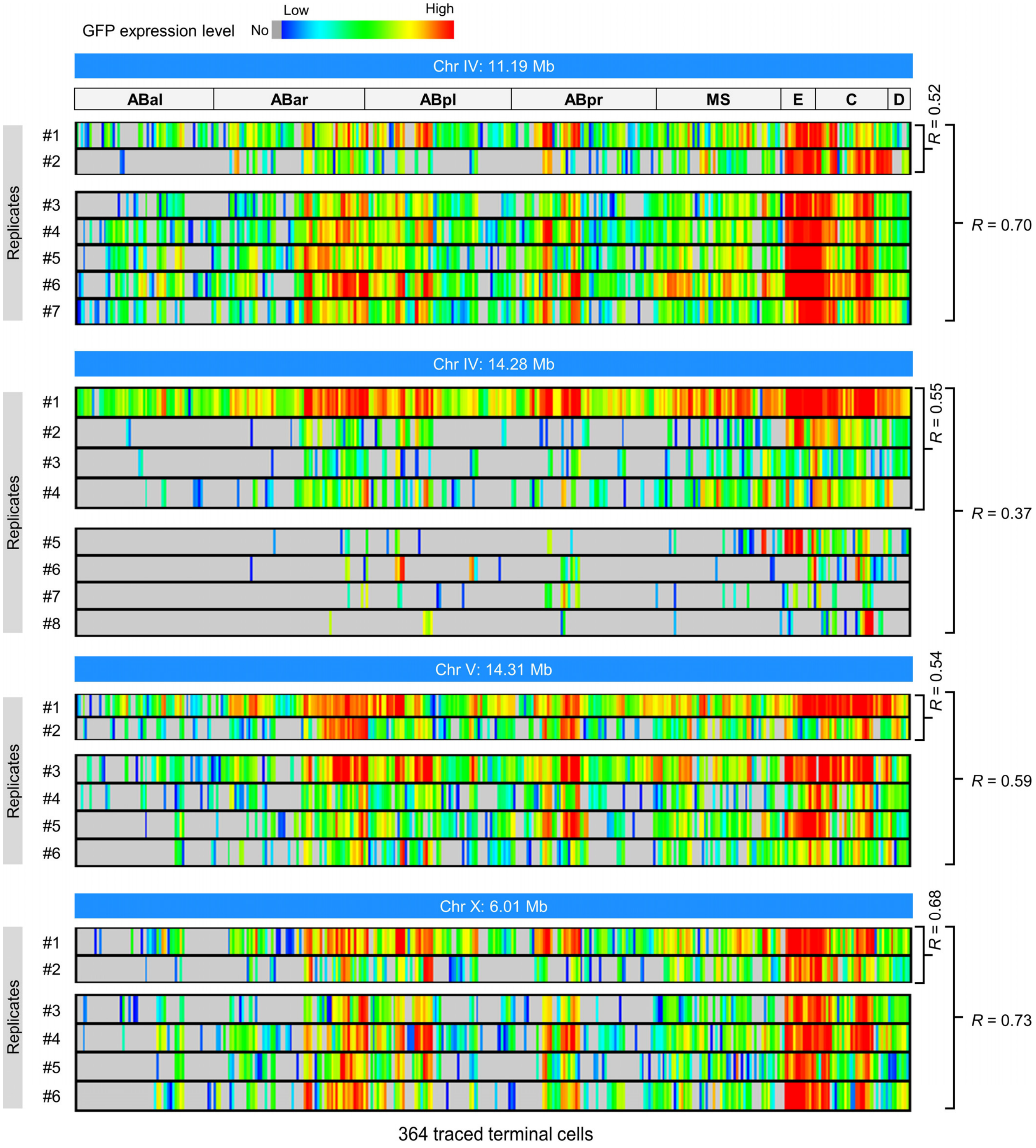

GFP expression level
No
Low
High
Chr IV: 11.19 Mb
ABal
ABar
ABpl
ABpr
MS
E
C
D
Replicates
#1
#2
#3
#4
#5
#6
#7
R = 0.52
R = 0.70
Chr IV: 14.28 Mb
#8
R = 0.55
R = 0.37
Chr V: 14.31 Mb
R = 0.54
R = 0.59
Chr X: 6.01 Mb
R = 0.68
R = 0.73
364 traced terminal cells

Appendix Fig S2. Expression of P*eef-1A.1*::GFP exhibits strong variability between experimental replicates at certain integration sites.

Each heatmap shows cellular expression levels (color gradient) of P*eef-1A.1*::GFP at four integration sites (indicated above the heatmap) in two batches of experimental replicates. Cells are ordered by lineage. Replicative embryos are grouped by batch, with the Pearson correlation coefficient of GFP expression between replicates shown on the right.

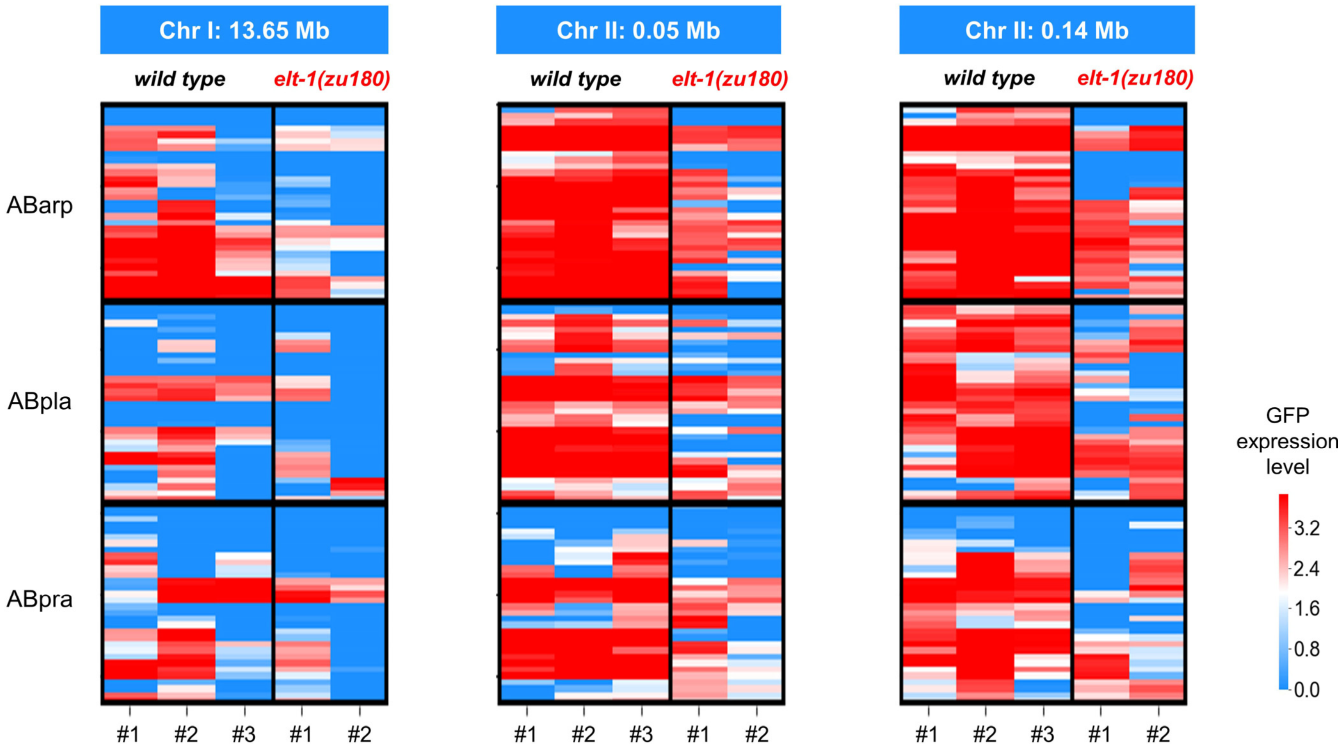

Appendix Fig S3. Chromatin activity landscape is coupled to tissue fate.

Comparison of cellular expression of GFP for three integration sites in cells from three major skin cell lineages upon perturbing the skin fate specifier. Each heatmap shows the expression level of GFP integrated into a specific position in cells from the ABarp, ABpla, and ABpra lineages, which normally differentiate into skin in all analyzed wild-type and *elt-1(zu180)* embryos. Each row is a cell and each column is an embryo.

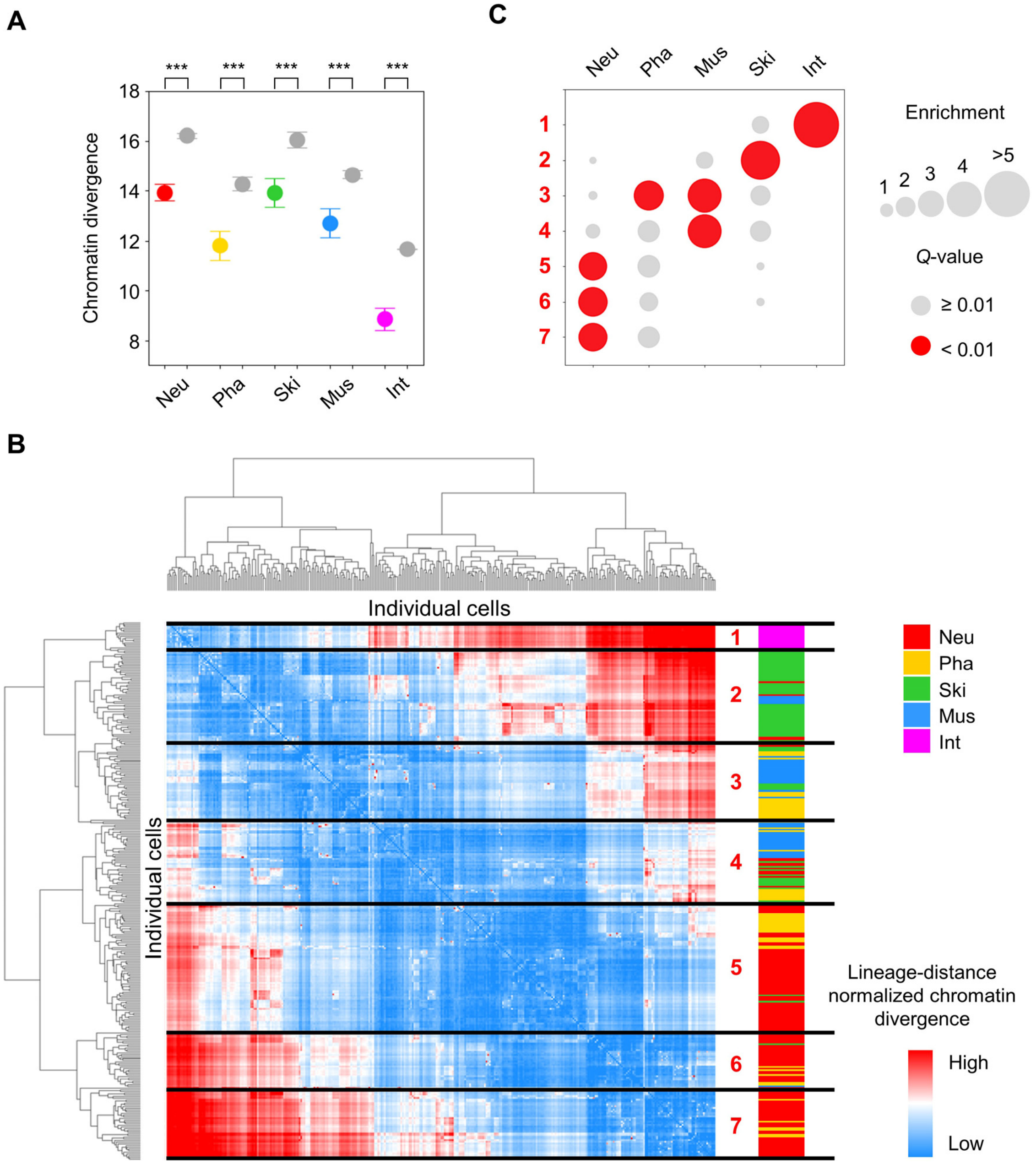
A
C
B
Chromatin divergence
18
16
14
12
10
8
***
Neu
Pha
Ski
Mus
Int
Enrichment
1
2
3
4
>5
Q-value
≥ 0.01
< 0.01
Individual cells
Lineage-distance normalized chromatin divergence
High
Low
1
2
3
4
5
6
7

Appendix Fig S4. Chromatin activity landscape is associated with tissue fate after controlling for the influence of cell lineage.

A. Chromatin activity divergences (mean ± 95% CI) of intra-tissue cells and inter-tissue cells (gray) after controlling for the influence of lineage distance between cells. For each pair of intra-tissue cells, the mean chromatin activity divergence of all inter-tissue cells having identical cell lineage distance was used as the control. Statistics: Mann-Whitney U test.

B. Hierarchical clustering of cells using lineage distance-normalized chromatin activity divergences between cells. Cell clusters are numbered, and the barcode on the right indicates the tissue type of each cell. Detailed information for each cell in each cluster is provided in Table EV7.

C. Enrichment of tissue types in cells in each cluster. Statistics: Fisher's exact test, Benjamini-Hochberg adjusted *P*-value.

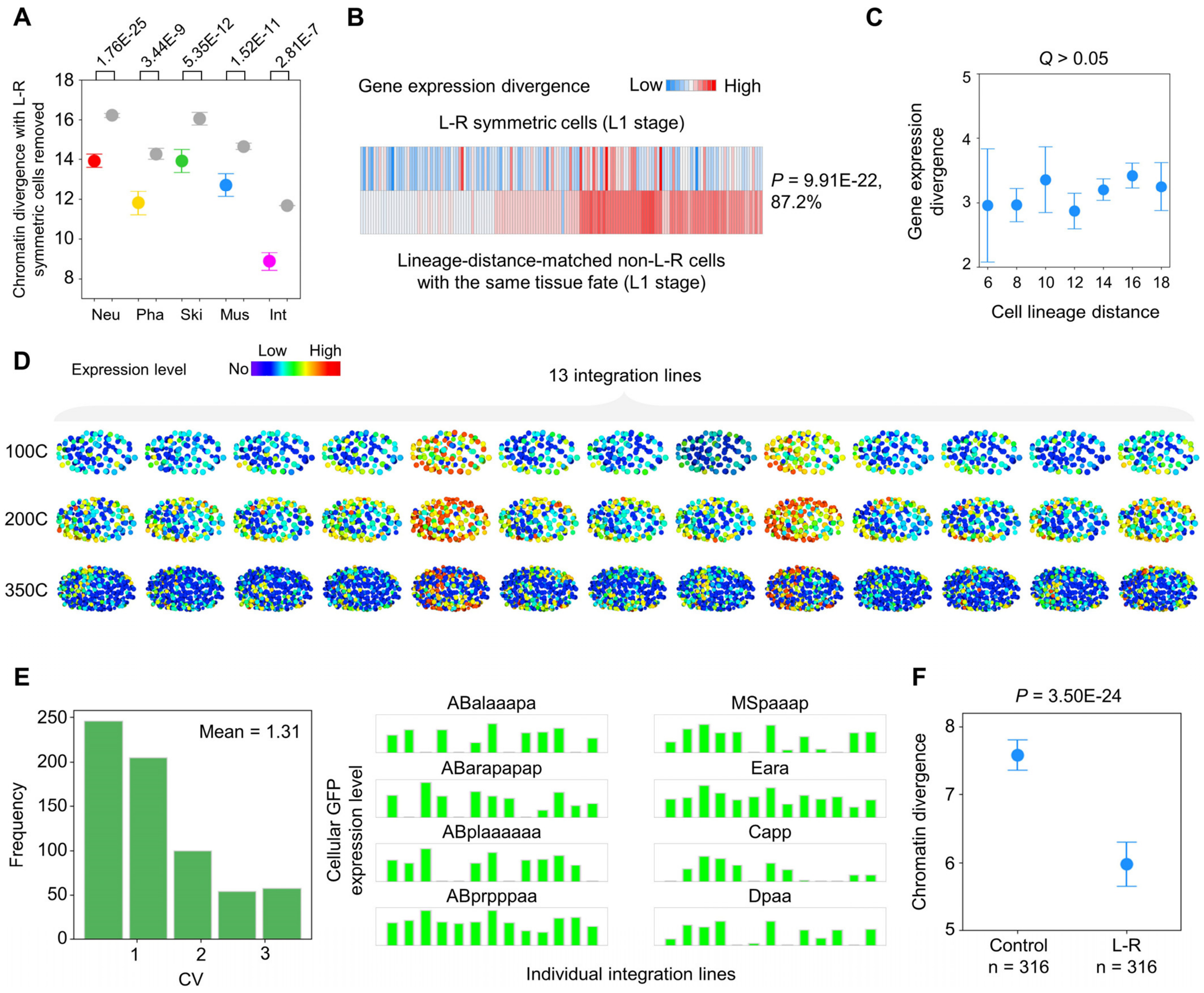

Appendix Fig S5. Predetermination of chromatin activity landscape during L-R symmetry establishment.

A. Chromatin activity divergences (mean ± 95% CI) of intra-tissue cells and inter-tissue cells (gray) after controlling for the influence of lineage distance and L-R symmetric cells. For each pair of intra-tissue cells, the mean chromatin activity divergence of all inter-tissue cells (excluding L-R symmetric cells) having identical cell lineage distance was used as the lineage-distance-matched control. Statistics: Mann-Whitney U test.

B. Heatmap visualization of gene expression divergence in L1-stage larvae between left and right cells in each pair of L-R symmetric cells/progenitors (top) and between lineage distance-matched non-L-R symmetric cells from the same tissue type (bottom). Statistics: Wilcoxon signed-rank test, n = 156.

C. Gene expression divergence between L-R symmetric cells with different cell lineage distances. Data are presented as mean ± 95% CI. Statistics: Pairwise Tukey-HSD Post-Hoc test, Benjamini-Hochberg adjusted P-value.

D. Position effects on P*nhr-2*::GFP::NLS expression at 13 genomic positions in all embryonic cells at three developmental stages. Each ellipsoid is a 3D rendering of a standardized embryo, with dots indicating embryonic cells and the color gradient indicating GFP expression level.

E. Left: Distribution of CV (coefficient of variation) of GFP expression across integration sites in the same cell. Right: Bar graph showing positional variation of GFP expression across 13 genomic positions in representative cells.

F. Comparison of chromatin activity divergences (mean ± 95% CI) between L-R symmetric cells and between lineage-distance-matched intra-tissue non-L-R symmetric cells (control) using the expression of P*nhr-2*::GFP::NLS. Statistics: Wilcoxon signed-rank test.
