# Supplementary material for: Single‐cell dynamics of chromatin activity during cell lineage differentiation in Caenorhabditis elegans embryos
Source: Mol Syst Biol. 2021 Apr 26;17(4):e10075. doi: 10.15252/msb.202010075 (PMC8073016; doi:10.15252/msb.202010075)
Supplement: Supplementary file 3 — Expanded View Figures PDF [file MSB-17-e10075-s008.pdf]

Expanded View Figures

Figure EV1. Quantification of position effects on GFP expression in single cells during *Caenorhabditis elegans* embryogenesis.

A Bar graph showing the number of traced time points (mean ± SD) for each lineaged cell across all analyzed embryos (n = 268). GFP intensities at each of these time points were measured and then averaged to represent cellular GFP expression level.

B Verification of the integration sites of reporter lines. Green boxes indicate the integration sites of all 113 strains examined in this study, and the 18 strains that were verified by PCR and sequencing are numbered with the sequences flanking the integration sites provided.

C Schematic of fluorescent intensity attenuation with depth. Due to different embryo orientations, the same cell in different embryos is located at a different depth relative to the microscope objective. DNO and VNO indicate the dorsal or ventral side of the embryo near the objective, respectively.

D Heatmap comparing cellular GFP intensity between experimental replicates of embryos with the identical or distinct orientation. Cells near and far from the center Z plane are grouped.

E Differences in cellular GFP intensity in multiple embryos of the same integration strains with different orientations were used to model the attenuation factor. Cells are ordered by lineage, and GFP intensities in replicates are grouped with red and green bars representing VNO and DNO embryos, respectively. The upper panel shows the raw results, and the lower panel shows the result adjusted by using an attenuation factor of 0.054 that exhibited the best performance.

F Representative examples showing the performance of attenuation correction. Scatter plots show the cellular GFP expression in embryos of the same integration strain with different orientations during imaging before (top) and after (bottom) the compensation of Z attenuation effect.

G Distribution of the average Pearson correlation coefficient of GFP expression levels (left) and consistency of the on/off expression patterns (right) between experimental replicates.

H Tree visualization of the fraction of integration sites at which the GFP is expressed in each cell.

© 2021 The Authors

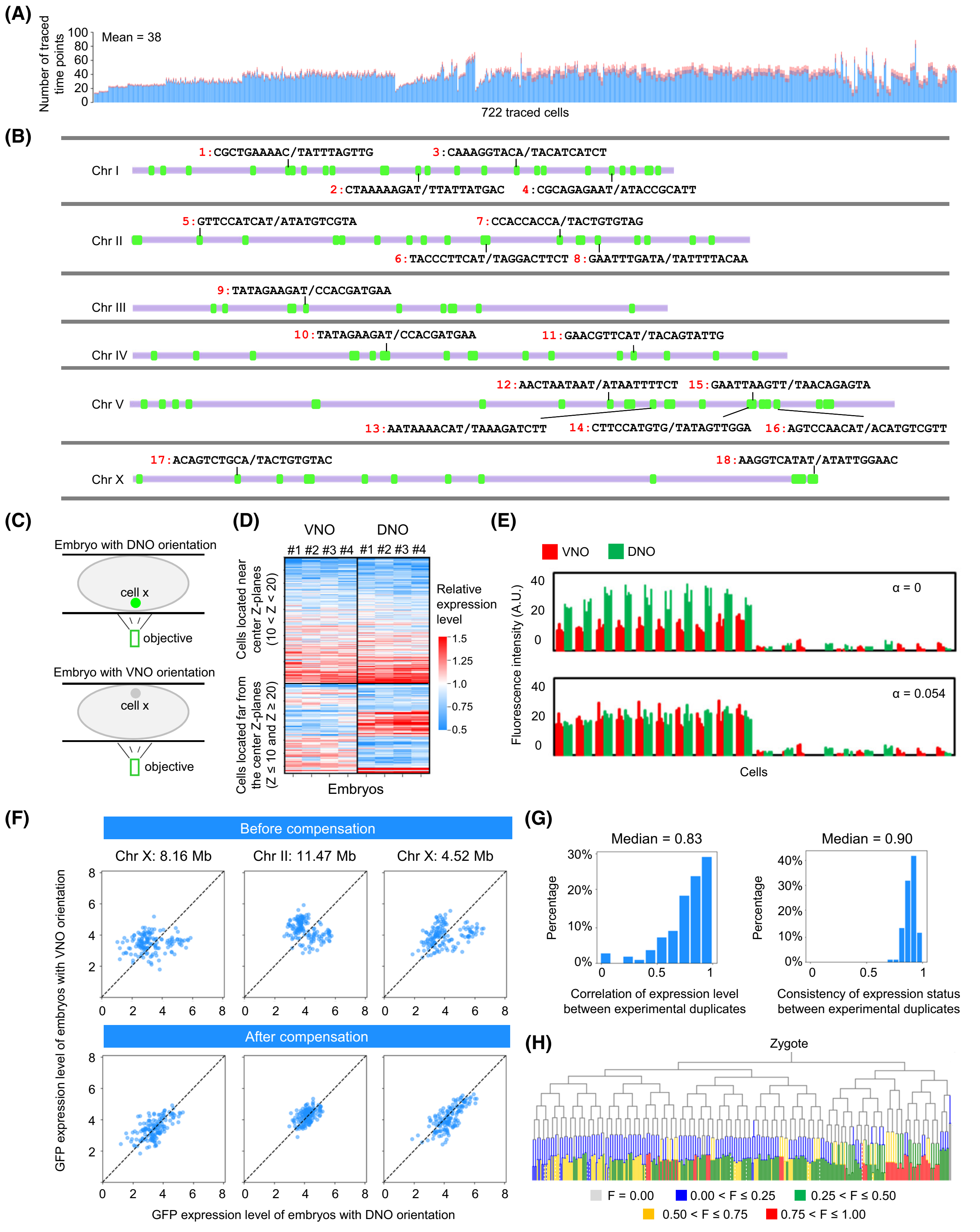

Figure EV1.

 © 2021 The Authors

Figure EV2. Position effects on GFP expression indicate chromatin activity.

A Comparison of average GFP expression levels across all traced terminal cells between autosomes ($n = 97$) and the X chromosome ($n = 16$).

B Comparison of average GFP expression levels between center ($n = 66$) and arm ($n = 47$) regions of chromosomes.

C Correlation of the observed average GFP expression levels with the values predicted by a combination of 19 types of histone modifications near the integration site ($n = 113$).

D Correlation of each of the 19 types of histone modifications with the average GFP expression levels across 113 genomic positions.

E, F Changes in cellular GFP expression integrated into multiple genomic positions after perturbing histone H3K9me3 (E) and H4K16ac (F). In each experiment, the top panel shows the micrograph comparison of the expression of ubiquitous mCherry and P*eef-1A.1*::GFP in all analyzed embryos, and the bottom panel shows the quantification results ($\log_2$ fold changes and Benjamini–Hochberg-adjusted *P*-values, Wilcoxon signed-rank test). Cell-by-cell comparisons of GFP expression levels in the 364 traced terminal cells were performed between *wt* and perturbed embryos. Scale bar = 10 µm.

G Comparison of average GFP expression levels between those located in the regions exhibiting active (states 1–3, $n = 12$) and silent (states 10–13, $n = 19$) chromatin states defined by Ho *et al* (2014).

H Comparison of average GFP expression levels between those located in the regions exhibiting active (states 1–5, $n = 14$) and silent (states 17, 18, and 20, $n = 26$) chromatin states defined by Evans *et al* (2016).

I Comparison of average GFP expression levels between accessible ($n = 39$) and non-accessible ($n = 74$) chromatin.

J Comparison of average GFP expression levels between LAD ($n = 57$) and non-LAD ($n = 56$) regions.

K Comparison of average GFP expression between intragenic ($n = 53$) and intergenic ($n = 60$) regions.

L Correlation of expression levels between GFP and endogenous genes in 5-kb (left) and 500-kb (right) intervals centered on the integration sites ($n = 89$ for 5-kb; 113 for 500-kb).

M Distribution of Pearson correlation coefficient between single-cell expression levels of GFP and endogenous genes located in a 500-kb interval centered on the integration sites. Only cellular transcriptomes that had been assigned with a unique identity ($n = 38$, left panel) and two possible identities ($n = 267$, right panel) were analyzed. Given that left–right symmetric cells generally exhibit very similar anatomy and function, we also treated a cell transcriptome as being assigned a "unique" identity if the assigned two identities are a pair of left–right symmetric cells. Chromatin activity landscapes of corresponding left–right symmetric cells were then averaged and compared with the scRNA-seq data (lower panel).

Data information: All inter-group statistics were performed by the Mann–Whitney *U*-test; all correlation statistics were performed by Pearson correlation. Box plot: The center band is the median, box limits are the first and third quartiles, box length indicates IQR, and whiskers either 1.5 times the IQR or the minimum/maximum value if it falls within a factor of 1.5 times of the IQR. Outliers not shown.

© 2021 The Authors

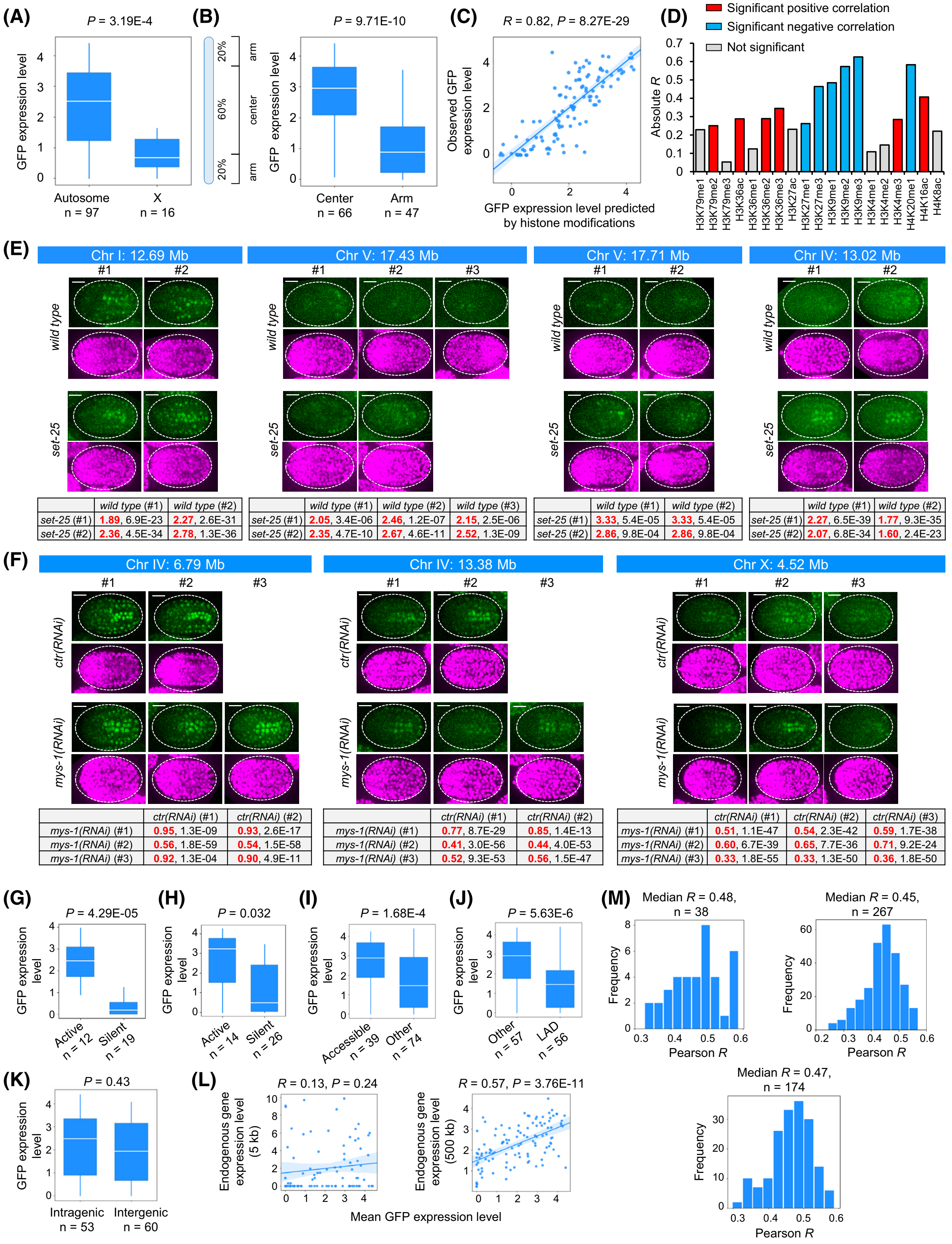

(E) Chr I: 12.69 Mb

	wild type (#1)	*wild type* (#2)
set-25 (#1)	**1.89**, 6.9E-23	**2.27**, 2.6E-31
set-25 (#2)	**2.36**, 4.5E-34	**2.78**, 1.3E-36

(E) Chr V: 17.43 Mb

	wild type (#1)	*wild type* (#2)	*wild type* (#3)
set-25 (#1)	**2.05**, 3.4E-06	**2.46**, 1.2E-07	**2.15**, 2.5E-06
set-25 (#2)	**2.35**, 4.7E-10	**2.67**, 4.6E-11	**2.52**, 1.3E-09

(E) Chr V: 17.71 Mb

	wild type (#1)	*wild type* (#2)
set-25 (#1)	**3.33**, 5.4E-05	**3.33**, 5.4E-05
set-25 (#2)	**2.86**, 9.8E-04	**2.86**, 9.8E-04

(E) Chr IV: 13.02 Mb

	wild type (#1)	*wild type* (#2)
set-25 (#1)	**2.27**, 6.5E-39	**1.77**, 9.3E-35
set-25 (#2)	**2.07**, 6.8E-34	**1.60**, 2.4E-23

(F) Chr IV: 6.79 Mb

	ctr(RNAi) (#1)	*ctr(RNAi)* (#2)
mys-1(RNAi) (#1)	**0.95**, 1.3E-09	**0.93**, 2.6E-17
mys-1(RNAi) (#2)	**0.56**, 1.8E-59	**0.54**, 1.5E-58
mys-1(RNAi) (#3)	**0.92**, 1.3E-04	**0.90**, 4.9E-11

(F) Chr IV: 13.38 Mb

	ctr(RNAi) (#1)	*ctr(RNAi)* (#2)
mys-1(RNAi) (#1)	**0.77**, 8.7E-29	**0.85**, 1.4E-13
mys-1(RNAi) (#2)	**0.41**, 3.0E-56	**0.44**, 4.0E-53
mys-1(RNAi) (#3)	**0.52**, 9.3E-53	**0.56**, 1.5E-47

(F) Chr X: 4.52 Mb

	ctr(RNAi) (#1)	*ctr(RNAi)* (#2)	*ctr(RNAi)* (#3)
mys-1(RNAi) (#1)	**0.51**, 1.1E-47	**0.54**, 2.3E-42	**0.59**, 1.7E-38
mys-1(RNAi) (#2)	**0.60**, 6.7E-39	**0.65**, 7.7E-36	**0.71**, 9.2E-24
mys-1(RNAi) (#3)	**0.33**, 1.8E-55	**0.33**, 1.3E-50	**0.36**, 1.8E-50

Figure EV2.

 © 2021 The Authors

Figure EV3. Chromatin activity dynamics across cell lineage correlate with lineage-coupled fate differentiation.

A Definition of cell lineage distance. Calculation of cell lineage distance (*D*) is shown for two examples in which the red circle indicates the lowest common ancestor (LCA) of the two target cells to calculate the lineage distance, with the number listing all cell divisions that lead to the two cells from the LCA.

B Schematic of comparing the developmental fate of progenitor cells. The developmental fate of a progenitor cell (black circle) is represented as the combinatorial pattern of tissue types (colored squares) it gives rise to following cell lineage tree. Fate divergence between two progenitor cells (*x* and *y*) was quantified by first determining whether the tissue fates are equal (score = 1) or not (score = 0) in each lineal equivalent terminal cell that was then averaged. In case, two progenitor cells produce different numbers of terminal cells, the smaller lineage (*y*) is expanded to match the larger (*x*) one, cells and tissue fates are expanded (stars) in the corresponding lineage branches.

C Changes in fate divergence (mean ± 95% CI) following the increase of cell lineage distance at 100-, 200-, and 600-cell stages (cell pair numbers from left to right: n = 51; 100; 192; 352; 640; 1,152; 1,024 for 100-cell stages; n = 95; 188; 368; 736; 1,568; 2,176; 5,632; 4,096 for 200-cell stage; n = 357; 567; 1,027; 2,010; 3,672; 7,263; 11,966; 24,872; 60,759; 31,110; 752 for 600-cell stage).

D Strategy to identify chromatin activity transition points. For each pair of daughter cells (DC1 and DC2) produced by a progenitor cell (gray circle), intra- (blue and red lines) and inter-daughter–lineage chromatin activity divergences (magenta line) were compared between terminal cells produced by the two daughter cells to determine whether the mother cell is a chromatin activity transition point.

E A representative chromatin activity transition point with numbers indicating corresponding inter- and intra-daughter–lineage chromatin activity divergences.

F Distribution of cellular transcriptome divergences between daughter cells following a cell division that exhibits chromatin activity transition. The blue line indicates the mean transcriptome divergence between daughter cells following non-chromatin-transition cell divisions. Statistics: Mann–Whitney *U*-test.

G Left: classification of traced terminal cells into 50 lineage groups. Figure showing the cell lineage leading to the progenitor cells of the 50 lineages. Right: Heatmap showing the average fraction of genomic positions exhibiting distinct on/off states of chromatin activity between cells in all pair-wise lineage comparisons. The value of intra-lineage comparison was subtracted from the value of the corresponding inter-lineage comparisons.

© 2021 The Authors

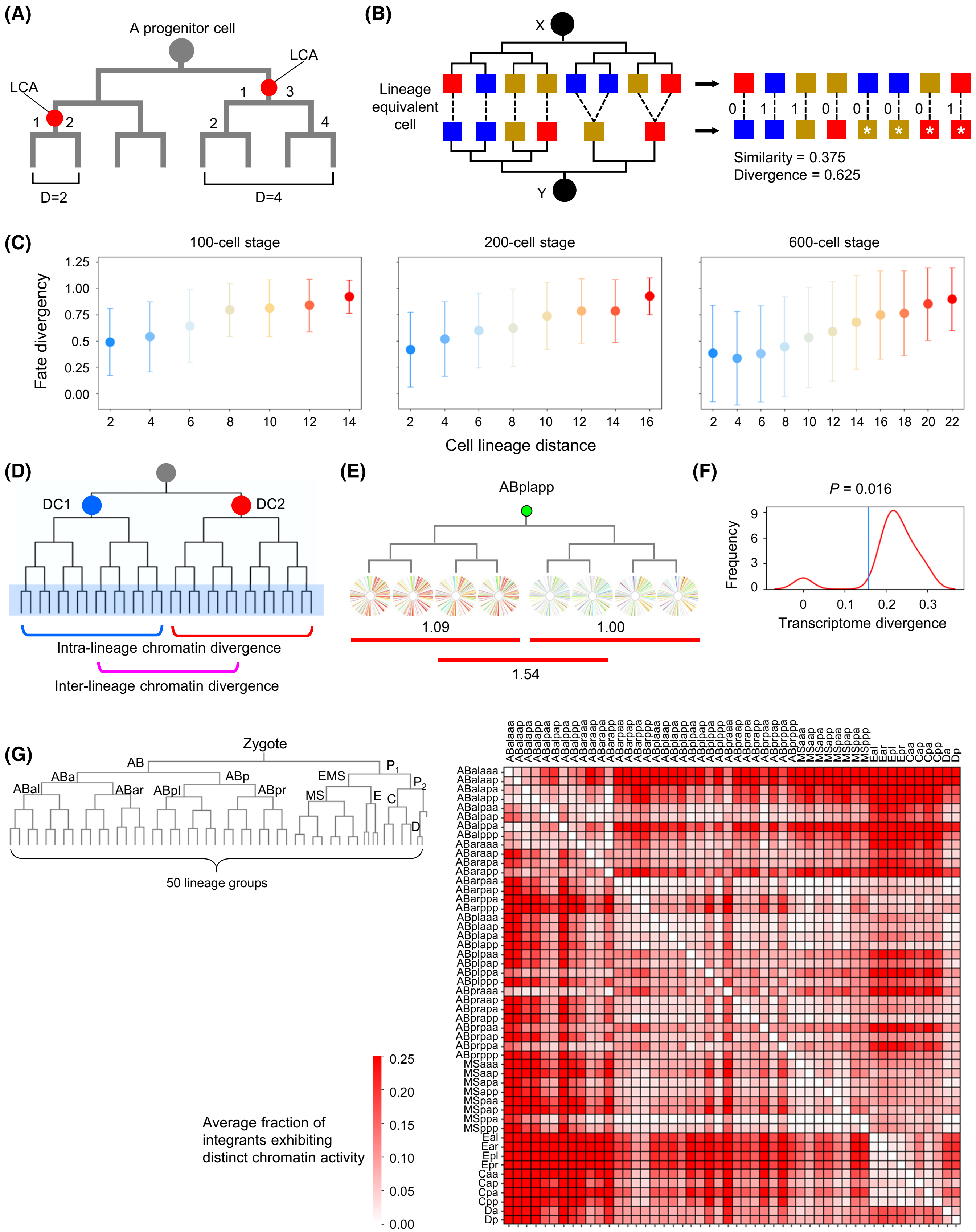

Figure EV3.

© 2021 The Authors

Figure EV4. Chromatin activity landscape is coupled to lineage fate.

A Cell lineage trees and characteristic programmed cell death (red cross) of the ABalp and ABara lineages in wild-type and *lag-1(RNAi)* embryos.
B A characteristic sequence of morphological changes in the nucleus was used to determine whether a cell (arrow) undergoes programmed cell death.
C Top: Each heatmap shows the relative expression levels of GFP (color gradient) integrated into a specific position in cells from the ABalp ($n = 32$) and ABara ($n = 32$) lineages in the wild-type and *lag-1(RNAi)* embryos. Cellular GFP expression levels were normalized to the mean value of cells from the ABarp lineage for both genotypes. Bottom: expression quantification results. Multiple embryos were analyzed for each genotype, and the ratio indicates how many times (wild-type-RNAi comparisons) the changes in GFP expression are consistent with the expectation. Statistics: Mann–Whitney *U*-test (***$P < 0.001$, NS, $P > 0.05$).
D Cell lineages and characteristic programmed cell death (red cross) in the EMS lineage of wild-type and *pop-1(RNAi)* embryos.
E Top: Each heatmap shows the relative expression level of GFP (color gradient) integrated into a specific position (indicated above) in cells from the MS ($n = 32$) and E ($n = 16$) lineages in the wild-type and *pop-1(RNAi)* embryos. Cellular GFP expression levels were normalized to the mean value of cells from the E lineage for both genotypes. Bottom: expression quantification results. The organization of the figure is the same as in (C).

Data information: All inter-group statistics were performed by the Mann–Whitney *U*-test; (***$P < 0.001$, NS, $P > 0.05$). Box plot: The center band is the median, box limits are the first and third quartiles, box length indicates IQR, and whiskers either 1.5 times the IQR or the minimum/maximum value if it falls within a factor of 1.5 times of the IQR. Outliers not shown.

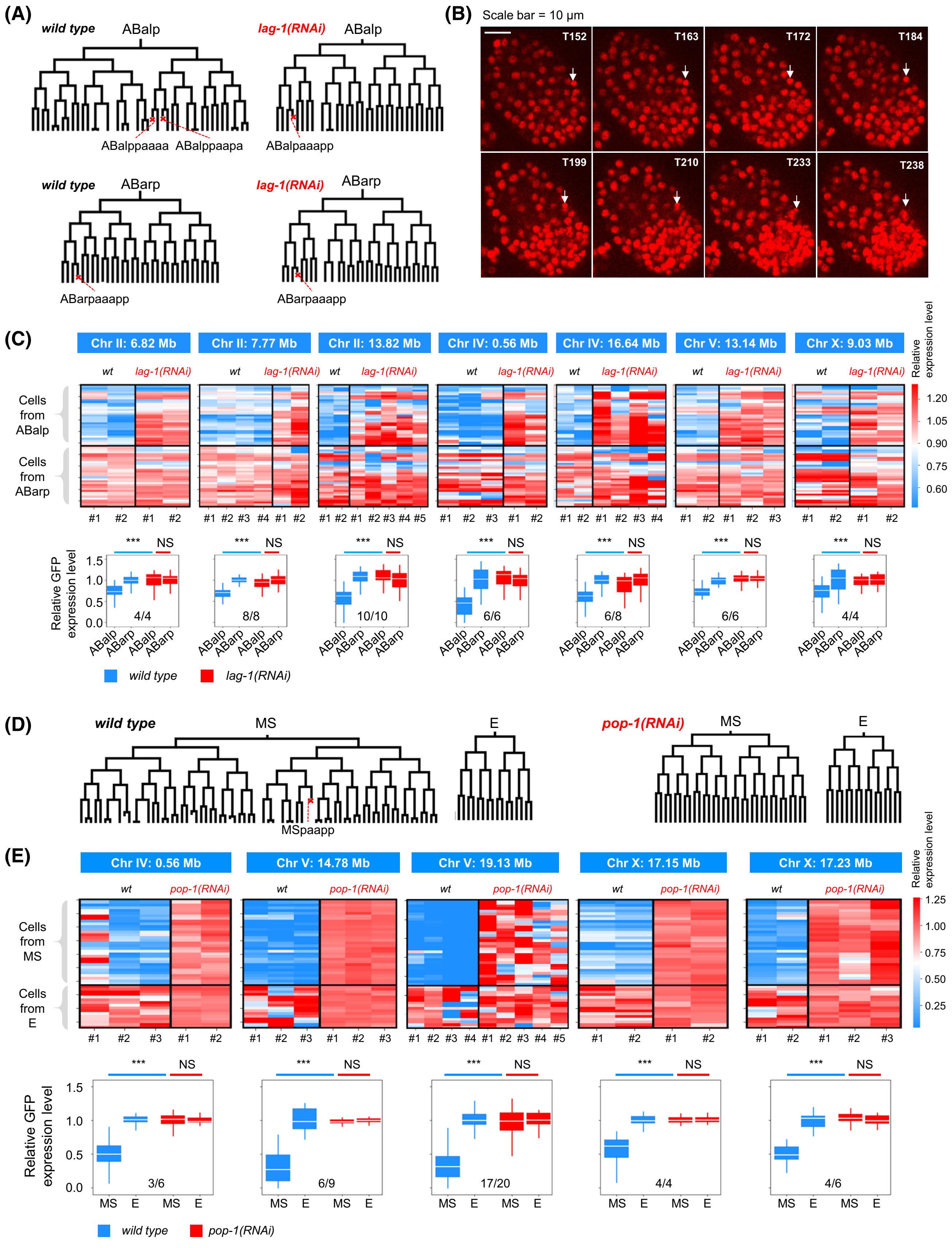

Figure EV4.

© 2021 The Authors

Figure EV5. Lineage-dependent heterogeneity in chromatin activity landscape and gene expression.

A Changes in chromatin activity divergences (mean ± 95% CI) following an increase in cell lineage distance between all post-mitotic intra-tissue cells (cell pair numbers from left to right: n = 28; 52; 72; 120; 286; 247; 704 for Ski; n = 5; 8; 6; 24; 48 for Mus; n = 4; 8; 18; 36 for Int). The neuronal system and pharynx were not included due to a small number of post-mitotic cells at the 350-cell stage. Chromatin activity divergence at certain cell lineage distances was not included because of a small number of eligible cells ($n < 3$).

B, C Comparison of cell lineage distances (mean ± 95% CI) between cells at the 350-cell (B, cell pair numbers from left to right: n = 1,382; 1,379; 1,382; 1,381; 1,379 for Neu; 287; 289; 284; 285; 286 for Ski; 157; 157; 154; 158; 154 for Mus) and 600-cell (C, cell pair numbers from left to right: n = 912; 913; 914; 912; 909 for Neu; n = 269; 266; 262; 265; 264 for Ski; n = 78; 72; 76; 76; 76 for Mus) stages having different transcriptome divergences. In many cases, two possible lineage identities were assigned to a cellular transcriptome, causing an inaccurate classification of cells based on lineage distance. We therefore classified transcriptome divergence between cells evenly into five bins and compared the average cell lineage distance between cells in each bin. The pharynx and intestine were not included because many of the cells were assigned more than two possible lineage identities, which would compromise the accuracy of the results.

D Changes in gene expression divergences (mean ± 95% CI) following the increase in cell lineage distance between cells in the L1-larvae stage (cell pair numbers: n = 18; 18; 33; 34; 95; 133; 48; 469; 598; 52 for Neu; n = 25; 50; 79; 133; 238; 222; 184; 419; 1,215; 16 for Pha; n = 26; 48; 68; 114; 266; 242; 669; 167 for Ski; n = 37; 68; 94; 204; 34; 355; 272; 896; 12; 8 for Mus; n = 8; 8; 18; 44; 16 for Int).

© 2021 The Authors

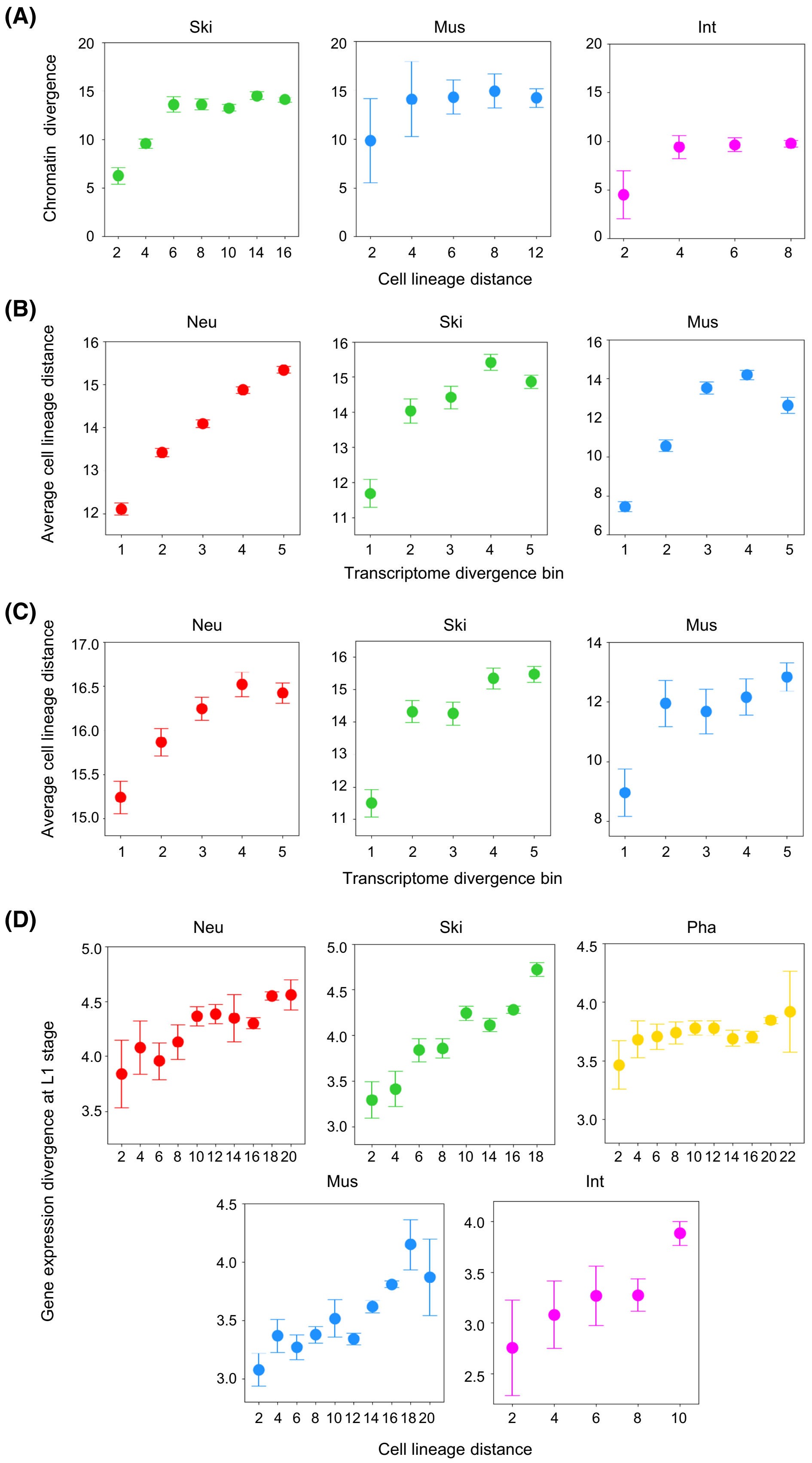

Figure EV5.

© 2021 The Authors
